# Recommended Drilling Parameters of Tungsten Carbide Round Drills for the Most Optimal Bone Removals in Oral Surgery

**DOI:** 10.1155/2018/3108581

**Published:** 2018-11-19

**Authors:** József Szalma, Ole Klein, Bálint Viktor Lovász, Edina Lempel, Sára Jeges, Lajos Olasz

**Affiliations:** ^1^Associate Professor, Department Head, Department of Oral and Maxillofacial Surgery, University of Pécs, 5 Dischka, 7621 Pécs, Hungary; ^2^Dentistry Student, Department of Oral and Maxillofacial Surgery, University of Pécs, 5 Dischka, 7621 Pécs, Hungary; ^3^PhD Student, Department of Oral and Maxillofacial Surgery, University of Pécs, 5 Dischka, 7621 Pécs, Hungary; ^4^Associate Professor, Department of Conservative Dentistry and Periodontology, University of Pécs, 5 Dischka, 7621 Pécs, Hungary; ^5^Professor, Faculty of Sciences, University of Pécs, 6 Ifjúság Street, 7624 Pécs, Hungary; ^6^Professor, Past President of the Hungarian AOMS, Department of Oral and Maxillofacial Surgery, University of Pécs, 5 Dischka, 7621 Pécs, Hungary

## Abstract

**Background:**

High temperatures during drilling can cause thermal osteonecrosis and abnormal wound healing. According to our best knowledge, a widely accepted recommendation for optimal drilling parameters in routine oral surgery bone removals does not exist.

**Purpose:**

Our aim was to investigate the correlations of different drilling parameters, including axial load and revolution speed on drilling temperatures and preparation times.

**Materials and Methods:**

Standard, 5 mm deep cavities were drilled in 20 PCF (lb/ft^3^) dens polyurethane blocks with 3 mm (50PCF) cortical layer using new and worn, 3.1mm in diameter tungsten carbide round drills. Worn drills were used in 50 impacted third molar operations before. Axial loads of 3N, 10N, and 25N and speeds of 4.000-8.000-16.000-40.000 revolutions per minute (rpm) were tested. Temperature differences of drilling parameters were calculated by 1-way ANOVA, followed by Tukey's HSD post hoc tests. Time differences and differences among “optimal” and “suboptimal” groups (with the cut-off value of 3°C and 3s) were estimated by Kruskal-Wallis test with pairwise comparisons. P<0.05 was considered significant.

**Results:**

The highest mean temperatures with new and worn drills were 4.64±0.53°C and 6.89±1.16°C, while drilling times varied between 0.16±0.02s and 22.77±5.45s. A 3°C and 3s cut-off value classified drillings significantly to (1) optimal [*3N and 8000-16000-40000 rpm or 10N and 4000-8000-16000-40000 rpm*] or suboptimal due to (2) high temperatures or (3) long preparation times. Using worn drills, the following parameters should be avoided: 3N with 4.000-8.000 rpm, 10N with 40000 rpm, and 25N at any revolutions.

**Discussion:**

The study extensively mapped the drilling temperatures and preparation times of tungsten carbide round drills. Temperatures did not exceed 10°C during drillings with maximal amount of cooling, as well as the drilling parameters, which kept temperatures and preparation times in the most optimal range which were clearly established.

## 1. Introduction

Bone removal is an important step in several oral surgical procedures. The most optimal bone removal is fast and painless and does not disturb normal healing processes. During drilling, excessive intraosseous heat production can lead to thermal osteonecrosis, which strongly influences the wound healing and regeneration mechanisms, and may lead, e.g., to alveolitis [1, 2]. According to Blum (2002), the incidence of alveolitis can be 25-30% after third molar removals, where drilling is a very frequent procedure [3]. The link between surgical trauma and dry socket is supported by several researchers, according to Noroozi and Philbert's comprehensive review [4]. Additionally, bone removal near to nerves (e.g., inferior alveolar nerve, incisal nerve) can reach such temperatures which have been reported to cause nerve conduction blocks [5].

However, the exact temperature is not known; a generally accepted threshold temperature causing osteonecrosis is the 47°C lasting for one minute [6, 7]. In addition, higher temperatures need much shorter period to be harmful (e.g., 50°C for 30 seconds [8]; 70°C for few seconds [9], or 90°C for few seconds [10]). Iyer et al. found that a temperature increase of only 4.3°C resulted in a significant worsening in the quality of newly formed bone [11]. Not only can high temperatures be harmful for nerves and bone tissues, but a 6°C rise in temperatures can also cause protein denaturation, alveolar bone resorption, and ankylotic reactions in periodontal ligaments [12]. Moreover, temperature increases above 3°C have been shown to cause changes in OPG/RANKL expression ratios in PDL fibroblasts [13].

Basically, two main groups of factors determine the heat production during drilling [1, 14]:* drilling parameters* including drilling speed, axial load, an existing predrilling, drilling depth, and the method of irrigation (external, internal, combined, and temperature of the cooling) and* drill specifications* including the drill's material, diameter, design of the drill (cutting face, flutes and helices, and drill point), and drill wear. In a recent investigation, we showed that the current tungsten carbide round drills after 30 coronectomies can produce intraosseous temperatures higher than 70°C [8000 revolutions per minute (rpm), 25 Newton (N), and 20 ml/min cooling] in pig ribs. Temperatures over 47°C lasted for up to 40 seconds [2]. Bone density is also an important factor, since drilling the cortical produces significantly more heat, than drilling in the spongiform bone [1, 7, 15].

In the field of oral implantology, usually very exact drilling protocols are published, and surgeons can set exact revolution speeds and apply recommended axial load according to a widely accepted agreement. However, during average universal oral surgical bone removals (usually performed by round drills), clinicians cannot lean on such guidelines or evidences. According to our best knowledge, there is no literature data or manufacturer's recommendation, on which revolutions and axial load values result in an optimal oral surgical bone removal, when tungsten carbide round drills are used.

The aim of this study was to determine the revolution speeds and axial load values which maintain a fast bone removal, but simultaneously cause acceptable intraosseous maximum temperatures in case of tungsten carbide round drills. Further aim was to find and show clinicians the significant differences between the new and worn drills, which were used 50 times in surgical third molar removal approaches.

## 2. Materials and Methods

### 2.1. Experimental Set-Up

Tungsten carbide round drills (HM141A, Hager & Meisinger, Neuss, Germany) with a diameter of 3.1mm were tested. We tested 4000, 8000, 16000, and 40000 revolutions per minute (rpm) speeds. Axial loading values were 3N, 10N, and 25N. The possible combinations of the four revolutions and three axial load values resulted in a total of twelve experimental groups (i.e., 3N-4000rpm; 3N-8000rpm; 3N-16000rpm; 3N-40000rpm; 10N-4000rpm; 10N-8000rpm; 10N-16000rpm; 10N-40000rpm; 25N-4000rpm; 25N-8000rpm; 25N-16000rpm; 25N-40000rpm).

**Figure 1:**
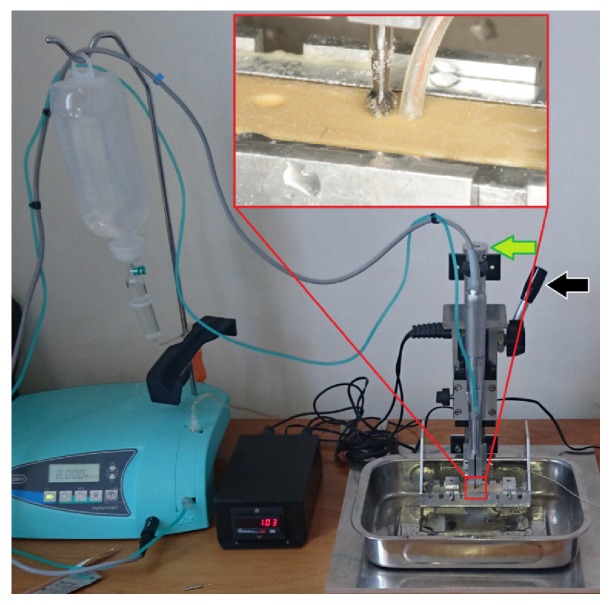
During the experiment, a custom made “drilling tower” was attached to a physio-dispenser unit and a surgical handpiece. After setting the bone surface contact (with a screw indicated by the green arrow), the use of the switch-lever (black arrow) allowed the handpiece to begin the axial movement and simultaneously started the time measurement, while another induction switch stopped the time measurement when the predetermined 5 mm depth was reached. Temperatures were measured by thermocouple probes connected to a registration unit. The thermosensor probe was embedded into rubber isolation tube (see magnified square) to eliminate cooling liquid's disturbing effects.

For irrigation, room temperature physiologic salt was used, and the amount was set to maximum (~60mL/min) during the entire experiment. A special testing device was able to standardize the following drilling parameters: revolution speed, axial load, and drilling depth, while measuring drilling times automatically [2]. The device was attached to a physiodispenser unit (Implantmed SI-915, W&H, Bürmoos, Austria) and a surgical straight handpiece (SL-11, W&H) (*Figure 1*).

Before drillings, the drills were positioned to a light “bone” surface contact. Then the drill was activated with the foot-pedal and immediately the operator released the switch-lever (indicated with the black arrow in Figure 1) which fixed the moving part, holding the handpiece. In the moment of releasing the switch-lever, a magnetic induction switch started the time measurement, which was stopped by another magnetic induction sensor automatically, when the drilling reached the 5 mm depth.

**Figure 2:**
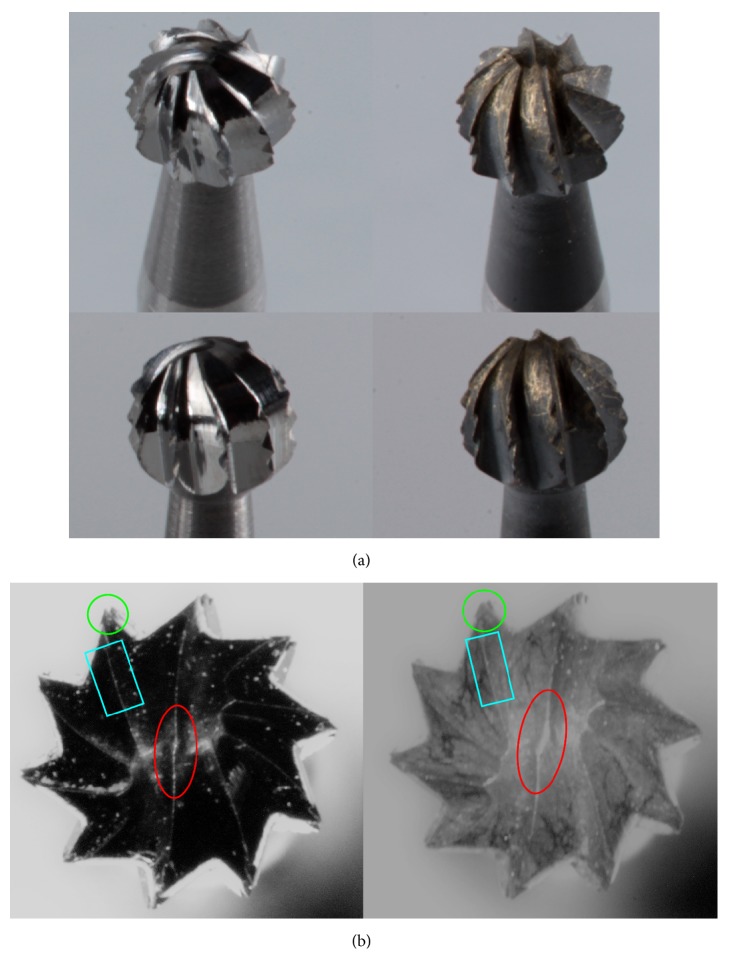
(a) Two of the tested tungsten carbide round drills in this study. Left: new drills and right: worn drills after 50 usage and sterilizations. Visualizing the signs of wear of the cutting edges is difficult without magnifications. (b) Left: new drill and right: worn drill. With the help of an operation microscope (~30x magnification), it is apparent that on the worn drill the cutting edges are visibly blunter. Cutting edges of the worn drill appear as a surface, rather than a narrow line (see colored squares and ellipses), while at the tip, as seen on the cross-cuts, it is more rounded (green circles).

From the combination of all drilling speeds and axial loads each having 8 drillings resulted in altogether 96 test cavities (4x3x8), followed by the same with the worn drills (additional 96 test cavities). The worn drills were used in 50 lower impacted third molar surgical procedures before and had 51 sterilization cycles. The macroscopic appearance of the new and worn drills was documented (*Figure 2*).

To eliminate the unwanted effect of wearing when testing the new drills, after eight drillings, another new drill was introduced; i.e., in each of the twelve groups (3N-4000rpm; 3N-8000rpm; 3N-16000rpm; 3N-40000rpm; 10N-4000rpm; 10N-8000rpm; 10N-16000rpm; 10N-40000rpm; 25N-4000rpm; 25N-8000rpm; 25N-16000rpm; 25N-40000rpm) a new drill was applied.

Drillings were performed in 20 PCF (pounds per cubic feet= lb/ft^3^) dens polyurethane (PU) blocks covered by 3 mm (50PCF) cortical layer [No. 1522-440, Sawbones Europe AB, Malmö, Sweden].

### 2.2. Temperature Measurements

**Figure 3:**
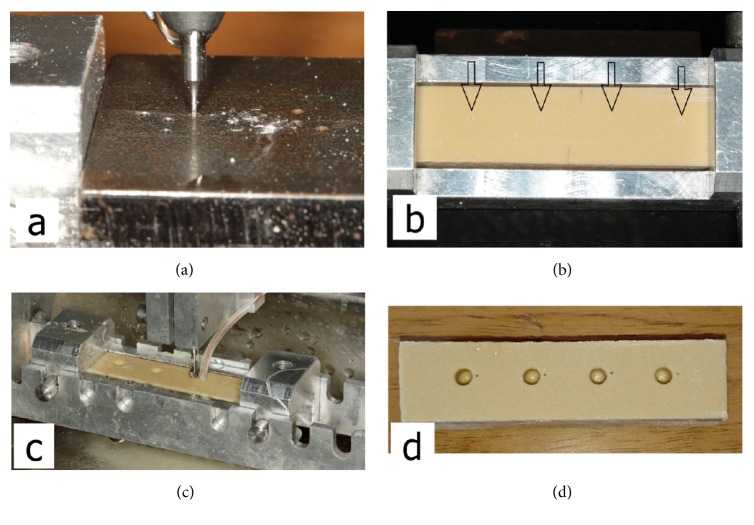
(a) The metal template is attached on the bone fixation box and determines the correct localization of the cavities for placing thermocouple probes. (b) Black arrows indicate thermocouple holes in the polyurethane sample. (c) The bone fixing box in one of the predetermined drilling places of the testing device. (d) After the drillings, the block confirms the correct positions of the test cavities and thermocouple holes.

PU specimens were fixed in a metal box. A metal drilling template was placed on this box to determine the locations of the thermocouple probes at the same distance (0.5mm) and depth (5mm) relative to the later drilled test cavities. 0.5mm in diameter Cu/CuNi thermocouple probes (K type, TC Direct, Budapest, Hungary) with an attached registration device (EL-EnviroPad-TC, Lascar Electronics Ltd., Salisbury, UK) characterized by 1 measurement per second frequency and 0.1°C resolution was used to gain intraosseous temperature data. To eliminate the influence of the cooling liquid, probes were covered by rubber tubes (prepared from 22-G wing needles, B. Braun Melsungen, Melsungen, Germany) during drillings (*Figures 1 and 3*). The ambient temperature was standard (air conditioned room, set to 24°C).

### 2.3. Statistical Analysis

The statistical analyses were performed with SPSS v. 23.0 (SPSS, Chicago, IL). The Kolmogorov-Smirnov test was applied to test the normality of the distribution of the data. To compare temperature data among different axial load and revolution speed groups, one way ANOVA was applied, followed by Tukey's HSD post hoc tests. To calculate the heat and preparation time differences among the subjectively defined “optimal” and “suboptimal” groups simultaneously, the nonparametric Kruskal-Wallis test (with pairwise comparisons) was used, since the distributions of the time data were found not normal. The same method was used to analyze the differences between average times, which were necessary to reach baseline temperatures. P values below 0.05 were considered significant.

## 3. Results

**Figure 4:**
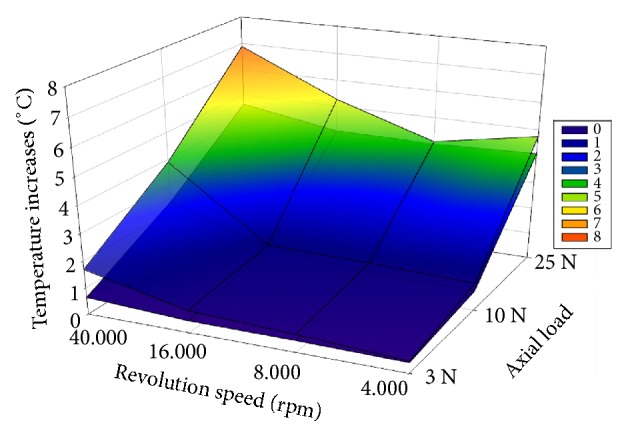
Three-dimensional plots of average maximum temperature values, measured by new and worn drills at the examined axial load values and revolution speeds. Transparent superficial plot: worn drills; lower plot: new drills.

**Figure 5:**
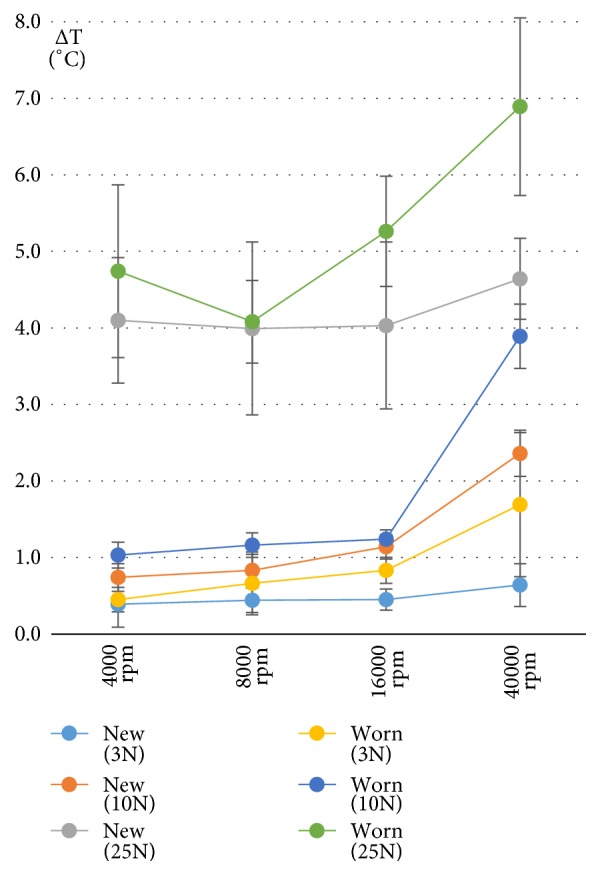
Line scatter diagram (with standard deviations) of average maximum temperature values, measured by new and worn drills at the examined axial load values and revolution speeds.

Regarding temperature data, it can be seen that none of the drillings reached the classic threshold temperature value of 47°C (*Figures 4 and 5*). However, according to Iyer's threshold level of 4.3°C, both new and worn drills approximated or exceeded this value at 25N of axial load.*Table 1* shows the significant differences regarding each revolution and axial load groups separately. In the case of new drills, only the axial load of 10N showed sufficiently different temperature elevations between 16000 and 40000 rpm revolutions. In addition, at each revolution speed, temperature values were significantly higher at 25N, than at lower axial loads. Only the 40000 rpm showed differences in maximum temperatures between the 3N and 10N axial load values. In the case of worn drills, the significantly highest temperature increases for each of the examined axial load values were seen at 40000 rpm. Highest temperatures were seen at 25N of axial load, while between 10N and 3N loads, temperatures were different only at a revolution of 40000 rpm. In addition, drillings at 3N showed clearly that, at each revolution speed, for both new and worn drills, the average temperature increases remained below 2°C.

**Figure 6:**
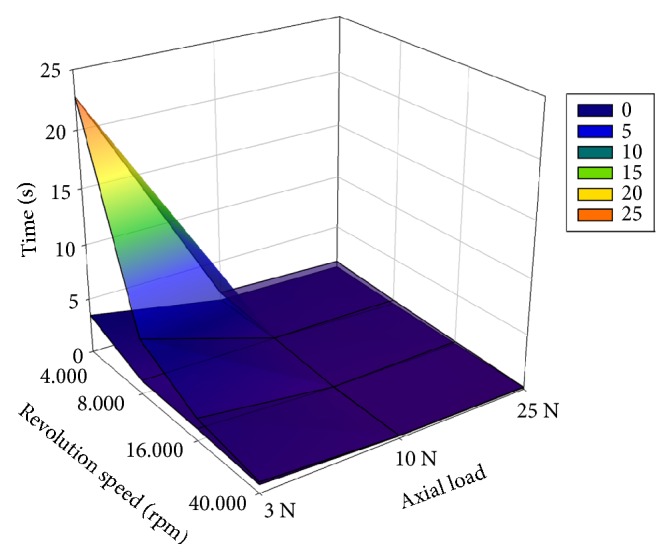
Three-dimensional plots of average drilling times observed by new and worn drills at the examined axial load values and revolution speeds. Transparent superficial plot: worn drills; lower plot: new drills.

**Figure 7:**
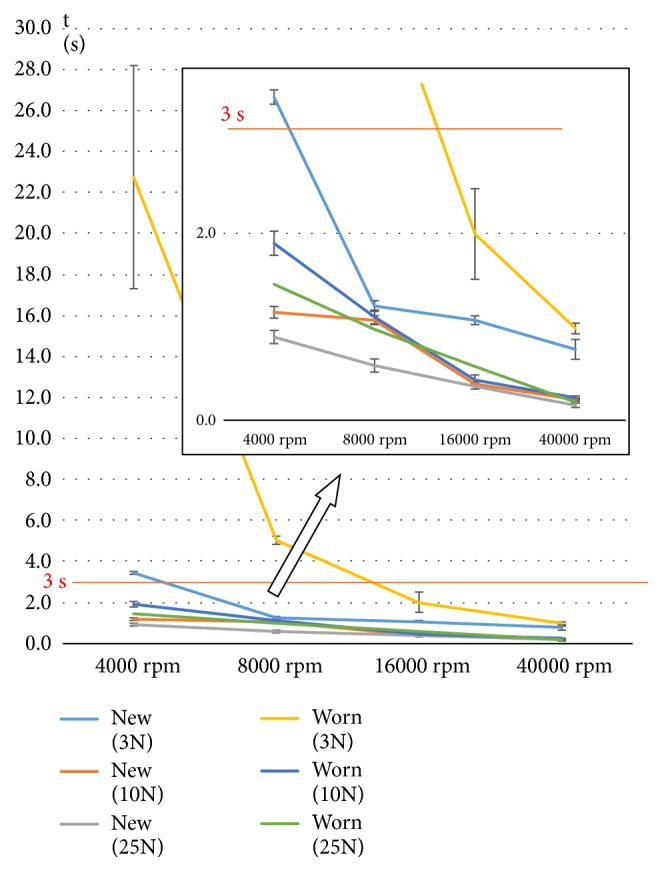
Line scatter diagram (with standard deviations) of average drilling times observed by new and worn drills at the examined axial load values and revolution speeds.

Regarding drilling times, there were approximately ~5x-20x differences between the slowest and fastest revolutions at the same axial load (Figures 6 and 7). At 40000 rpm, the gap between the preparation times of new and used drills was minimal. When we chose individually the threshold value of 3 seconds for a clinically acceptable drilling (for an only 5 mm deep cavity it seems realistic), data indicated that drilling with worn drills at 3N and with the two slowest revolutions resulted in much longer drillings (6 and 22.5 s versus ≤ 3 s). During those slow drillings, the temperature increases were maximally acceptable.

The average intervals in which temperatures returned to the baseline showed that only the drillings with worn drills at 25 N resulted in longer periods than 30 seconds. But the times of reaching the baseline were very different at 25 N, as well (p<0.001; Kruskal-Wallis test). At 4000 rpm (92±14.41 s), the time it took for the temperature to decrease to baseline was comparable to 8000 rpm (60.4±3.85 s), but significantly longer than at 16000 rpm (40.8±2.77 s; p=0.045) and 40000 rpm (29.2±4.32 s; p<0.001). The time difference between 8000 and 40000 rpm was also significant (p=0.045) but between 16000 and 40000 rpm it was not. The average durations where temperatures reached the initial values in case of 3N and 10N axial load (at every investigated revolution speed) were between 11 and 26 seconds. Accordingly, worn drills at 3 N axial load and 4000 or 8000 rpm resulted in extremely long preparations, while at 25 N load it took the longest for the temperature to return to the initial baseline.

**Figure 8:**
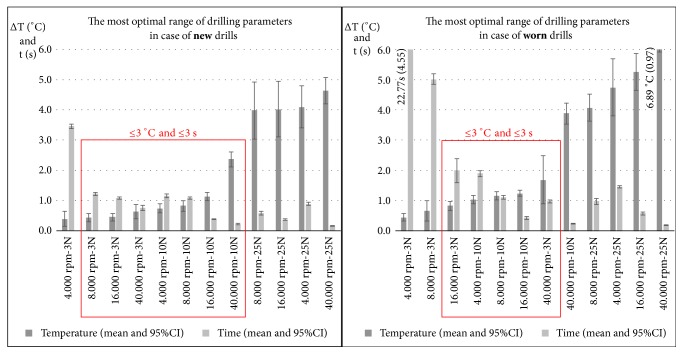
The drilling parameters at which the most optimal intraosseous heat and preparation times (red squares) were observed by new and worn drills.

When we subjectively defined the cut-off values of 3°C temperature increase and 3-second preparation time as the highest values for most optimal drilling parameters (*Figure 8*), statistically significant differences were found between the so-called “optimal” and “suboptimal” groups regarding both temperatures and drilling times (*Table 2*).

## 4. Discussion

During oral surgical bone removals, the surgeons' aim is to ensure fast drilling, low temperatures, and an optimal wound healing by choosing the right drill specifications and drilling parameters. This research aimed to map intraosseous temperatures and preparation times on different revolution speeds and axial loads at simulated oral surgical bone removals to find the most optimal drilling parameters. According to our best knowledge, this is the first attempt to investigate tungsten carbide round drills at different revolutions and axial load values, with the intention of observing their performance in simulated oral surgical circumstances.

During drilling, heat arises due to three mechanisms: (1) shear deformation of the shear zone, (2) friction between the rake face and the cut chip, and (3) friction between the flank face and the new bone surface [14, 16]. Revolution speed and axial pressure are important determinants of heat generation in the bone [1, 17]. While increasing independently the speed or the load results in increased temperatures, when increasing both together a more efficient drilling is seen without significant increase in temperatures [16]. The current results cannot confirm the above-mentioned observation, since the highest temperatures were observed at the highest revolution speed and axial load (*Figures 4 and 5*). It is important to note that our study used very different geometry drills (round drills versus spiral drills).

Literature data shows in agreement that temperatures increase with an increasing drilling speed, up to 10000 rpm [1, 14, 16]. This was seen in one of our earlier study also, where during mini-implant predrilling with spiral drills drilling at 100 or 200 rpm produced significantly less heat than at 1200 rpm (≤3°C versus 12.3°C) [18]. In addition, the current investigation showed a further significant temperature rise between 16000 and 40000 rpm drillings.

In another investigation, where the drilling speed was kept constant (49000 rpm) and axial load was increased from 1.5N to 9N, it was observed that heat productions increased first, and after 4N they decreased [19]. As the axial load was increased, the temperatures increased constantly; however the preparation time reduced after 4N, which resulted in reduced total heat productions [19]. This observation cannot be commented correctly, because only one axial load value was in the above range, while our highest axial load was significantly higher. Indeed, the average time period in which temperatures returned to baseline after reaching the maximum was the longest at 25 N axial loads in our study; however this value was only significant in the case of worn drills.

Our highest axial load resulted in very short drilling times but caused the highest temperature increases. At worn drills, another important observation was that 25 N loads resulted in significantly the longest intervals temperatures which were above the baseline. It is important to note that an excessively increased feeding rate can cause also microcracks in the bone or the breakage of the drill bit [20, 21] or can lead to drill-bit breakthrough [14]. The combination of the high axial load and low drilling speed causing microcracks was not investigated in our study.

Iyer et al.'s histological studies showed that high speed drilling (~400000 rpm) resulted in higher rate of bone healing and better quality of new bone formation, compared to low (2000 rpm) or intermediate speed (30000 rpm) drillings [11, 22]. Reingewirtz et al. showed that temperatures rose at speeds in the range of 400 rpm–7000 rpm, decreased in the range of 7000 rpm–24000 rpm, and stayed constant in the range of 24000 rpm–40000 rpm [23]. In contrast, our results showed that at 3N and 25N temperatures were similar as revolution speed was increased; however at 10N, there was a bigger temperature rise between 16000 and 40000 rpm. It is very important to note again that, in general, literature data characterizes spiral drills and not the round drills of our study. Furthermore, the differences in drilling parameters of different medical disciplines allow only limited comparisons. In the oral cavity axial pressures during drillings are usually under 30N, while in traumatology or orthopedics it can be as high as 80-200N [16].

Drill wear is a commonly discussed topic regarding drilling [24]. Researchers usually find a direct correlation between drill wear and intraosseous temperatures. While some authors found significant temperature increases and signs of drill wear after 25-40-50 usages of the drill [25–28], others found it only after 100 [29] or 600 [30] usages. Our earlier study, investigating the same drills, indicated clearly that, after 20 and 30 coronectomies, drills show significant signs of wear (i.e., fractured cutting edges, missing cross cuts), while temperatures (2.2-3.3x) and drilling times (5.3-12.5x) increased significantly compared to the new drill [2]. In contrast, our other investigation using mini-implant predrills showed temperature increases only after 150 usages [18]. Additionally, Marenzi et al. (2018) identified surface micromorphology of the drills as an important factor which may contribute to intraosseous heat productions [24]. The present study showed that after 50 drillings and concurrent sterilization cycles, detecting the clear, unambiguous signs of wear without magnification is almost impossible; however in some drilling groups drills produced significantly higher temperatures and/or slower preparations.

The study had some limitations as well. The current in vitro bone simulation model may show different thermal characteristics as living human bone; however, polyurethane is a frequently applied testing medium for such experiments [31]. In addition, while our in vitro experiment was able to control and keep drilling parameters constant, intraorally parameters are continuously changing and, e.g., axial load may reach higher or even extreme values. Moreover, intraorally the maximum amount (~60 ml/min) of irrigation may already disturb the patient, so according to the surgeons' choice or in some intraoral critical situations (retromolar area, disturbing effect of excessive soft tissues or the flap) a reduced irrigation volume can occur [14, 32]. With a reduced irrigation, maximum temperatures may be significantly higher [33].

## 5. Conclusions

Considering the maximum intraosseous temperatures and preparation times, the following recommendations can be given for oral surgical bone removals, when irrigation is set to the maximum level.

The most optimal parameters for drilling with new tungsten carbide round drills (Ø= 3.1mm) are an axial load of 3N with a speed of 8.000-16.000-40.000 rpm or an axial load of 10 N with 4.000-8.000-16.000-40.000 rpm revolutions. When axial load is higher, temperatures increase significantly, and the reduction in drilling times suggests no clinical benefits. With worn drills, the combinations of 3N axial load and 4000-8000 revolutions (long preparations), 10N axial load, and 40.000 rpm or a 25N axial load at any speeds should be avoided, because of possible harmful peak ∆ temperatures up to ~9°C. According to our results, the axial load had the biggest impact on temperature elevations, especially at 25N, independently from the applied revolution speeds, where the highest temperature increases were observed.

## Figures and Tables

**Table 1:** Temperature increases at different drilling parameters [°C (standard deviations)].

	Axial load	Revolution speed				
		4.000 rpm	8.000 rpm	16.000 rpm	40.000 rpm	*P value∗*
New drill	3 N	0.39(0.30)^a A^	0.44(0.16)^a A^	0.45(0.14)^a A^	0.64(0.28)^a A^	0.168
	10 N	0.74(0.18)^a A^	0.83(0.21)^a A^	1.14(0.16)^b A^	2.36(0.30)^c B^	**<0.001**
	25 N	4.10(0.82)^a B^	3.99(1.13)^a B^	4.03(1.09)^a B^	4.64(0.53)^a C^	0.473
	*P value∗∗*	**<0.001**	**<0.001**	**<0.001**	**<0.001**	

Worn drill	3 N	0.45(0.16)^a A^	0.66(0.41)^a A^	0.83(0.17)^a A^	1.69(0.94)^b A^	**<0.001**
	10 N	1.03(0.17)^a A^	1.16(0.16)^a A^	1.24(0.12)^a A^	3.89(0.42)^b B^	**<0.001**
	25 N	4.74(1.13)^a B^	4.08(0.54)^a B^	5.26(0.72)^a B^	6.89(1.16)^b C^	**<0.001**
	*P value∗∗*	**<0.001**	**<0.001**	**<0.001**	**<0.001**	

Abbreviations: rpm, revolutions per minute; N, newton.

*∗* Different small letters indicate statistical differences per lines (a, b, c). Tukey's HSD post hoc tests.

*∗∗* Different capitals indicate statistical differences per columns (A, B, C). Tukey's HSD post hoc tests.

**Table 2:** The statistical differences between optimal and suboptimal drilling parameter groups.

Drilling parameters		Temperatures	Time
**Optimal,** regarding temperatures & time	Mean	1.06^A^	0.97^a^
	N	96	96
	Std. Deviation	0.48	0.45

**Suboptimal,** because of temperatures	Mean	4.40^B^	0.57^b^
	N	72	72
	Std. Deviation	1.36	0.41

**Suboptimal,** because of time	Mean	0.54^C^	5.92^c^
	N	24	24
	Std. Deviation	0.28	8.02

Total	Mean	2.32	2.04
	N	192	192
	Std. Deviation	1.99	4.59

Abbreviations: different capitals and lower cases indicate statistical differences between means. Kruskal-Wallis test, pairwise comparisons, p<0.05.

## Data Availability

The data used to support the findings of this study are available from the corresponding author.
